# Characterization of Virulence Factors in Enterotoxin-Producing *Staphylococcus aureus* from Bulk Tank Milk

**DOI:** 10.3390/ani12030301

**Published:** 2022-01-26

**Authors:** Hye-Ri Jung, Young Ju Lee

**Affiliations:** College of Veterinary Medicine & Zoonoses Research Institute, Kyungpook National University, Daegu 41566, Korea; heelee1214@naver.com

**Keywords:** bulk tank milk, enterotoxin, virulence factor, bovine mastitis

## Abstract

**Simple Summary:**

*Staphylococcus aureus*, apathogen that causes bovine mastitis, produces various virulence factors, and human consumption of milk contaminated with the *S. aureus* enterotoxin may pose a public health risk. This study analyzed the genetic characteristics of bovine-mastitis-related virulence factors to evaluate the potential pathogenesis of *S. aureus* isolated from bulk tank milk. The results show that *S. aureus* isolated from bulk tank milk, not from mastitis, had a high prevalence of virulence factors and that the high presence of enterotoxins may be due to poor hygiene. Therefore, developing a strong monitoring and sanitation program for dairy factories is important to ensure hygienic milk production.

**Abstract:**

*Staphylococcus aureus*, a persistent mastitis-causing pathogen, produces various virulence factors, including enterotoxins. This study analyzed the genetic characteristics of bovine-mastitis-related virulence factors to evaluate the potential pathogenesis of *S. aureus* isolated from bulk tank milk. Among 93 *S. aureus* isolates from 396 dairy farms operated by 3 dairy companies in Korea, 40 (43.0%) isolates carried one or more enterotoxin genes. Moreover, *S. aureus* carrying enterotoxin genes showed a higher prevalence in all virulence genes tested in this study except for *pvl* and *lukM*, which were not detected in any isolate, than in the isolates without enterotoxin genes. In particular, the prevalence of six genes (*hla*, *hlb*, *lukED*, *fnbA*, *clfA,* and *clfB*) was significantly higher in *S. aureus* carrying the enterotoxin genes than in the isolates without the enterotoxin genes (*p* < 0.05). The most common multilocus sequence type of enterotoxin-producing isolates was ST188, and all isolates of ST188 harbored the *see* gene. *S. aureus* isolated from bulk tank milk, not from mastitis, had a high prevalence of virulence factors, posing a public health threat. Moreover, a high presence of enterotoxins in bulk tank milk is probably because of poor hygiene; therefore, it is important to develop strong monitoring and sanitation programs for dairy factories.

## 1. Introduction

*Staphylococcus aureus* (*S. aureus*) is one of the most common pathogens causing contagious mastitis in the dairy industry [1]. In particular, *S. aureus* is persistent and causes chronic mastitis, and consumption of these dairy products transmits virulence factors from the contaminated milk to humans and may pose a public health risk [2,3].

*S. aureus* has various virulence factors, such as toxic shock syndrome toxin-1 (TSST-1), enterotoxins, and leukotoxins. In particular, the staphylococcal enterotoxin, belonging to the superantigen family, is the most potent because it can induce polyclonal activation of T cells at picomolar concentrations [4]. This activity is suspected to enhance virulence by inhibiting the host response to staphylococcal antigens produced during infection or present during toxinoses. Moreover, ingestion of enterotoxins causes severe food poisoning with vomiting, nausea, and diarrhea [5]. To date, more than 20 types of enterotoxins have been identified, of which classical enterotoxin types (sea-see) pose serious public health concerns because they retain their biological and immunological activities after pasteurization [6]. Additionally, classical enterotoxins account for more than 90% of staphylococcal food poisoning cases worldwide [7], and some new enterotoxins (seg-sei) exhibit emetic activity [5].

Other virulence factors, such as adhesion and biofilm-related genes in *S. aureus*, can also cause various diseases in humans, ranging from mild skin infections to severe life-threatening infections. Moreover, Panton–Valentine leukocidin (PVL) disrupts the membranes of host defense cells and erythrocytes by the synergistic action of two specific proteins, lukS-PV and lukF-PV; therefore, it is also associated with bovine mastitis [8,9]. Although *S. aureus* was isolated from normal bulk tank milk, not from mastitis in this study, we analyzed the genetic characteristics of bovine-mastitis-associated virulence factors to evaluate the potential pathogenesis of *S. aureus*.

## 2. Materials and Methods

### 2.1. Bacterial Isolation

*S. aureus* was isolated from 1,588 batches of bulk tank milk from 396 dairy farms in 6 factories (A1, A2, B1, B2, B3, and C1) operated by 3 dairy companies (A, B, and C) according to standard microbial protocols published by the Ministry of Food and Drug Safety (2018) [10]. Briefly, 1 mL of the milk sample was inoculated in 9 mL of tryptic soy broth with 6% NaCl (BD Biosciences, Sparks, MD, USA). After incubation at 37 °C for 24 h, each medium was streaked onto 5% sheep blood agar (KOMED, Seoul, Korea). Confirmation of *S. aureus* was performed using PCR with a species-specific primer as described previously [11]. If two isolates of the same origin showed the same antimicrobial susceptibility patterns, only one isolate was randomly chosen. A total of 93 *S. aureus* isolates were tested for this study.

### 2.2. Detection of Virulence Factors

The presence of the genes encoding the enterotoxins (*sea*, *seb*, *sec*, *sed*, *see*, *seg*, *seh*, *sei,* and *sej*), the toxic shock syndrome toxin (*tsst-1)*, hemolysins (*hla* and *hlb*), Panton–Valentine leukocidin (*pvl*), leukocidins (*lukED* and *lukM*), fibronectin binding proteins (*fnbA* and *fnbB*), clumping factors (*clfA* and *clfB*), and intercellular adhesion (*icaA* and *icaD*) were detected by PCR using the Accupower PCR PreMix (Bioneer, Daejeon, Korea). The primers are listed in Table 1.

### 2.3. Molecular Typing

The genetic relationship of *S. aureus* with one or more enterotoxins was analyzed with multilocus sequence typing (MLST) and pulsed-field gel electrophoresis (PFGE). MLST was performed as previously described by Saunders and Holmes (2014) [20], and seven housekeeping genes (*arcC*, *aroE*, *glpF*, *gmk*, *pta*, *tpi,* and *yqiL)* purified using the GFX PCR DNA and Gel Band Purification Kit (Amersham Bioscience, Freiburg, Germany) were sequenced with an automatic sequencer (Cosmogenetech, Deajeon, Korea). Sequence types (STs) were obtained by combination using the *S. aureus* database (https://pubmlst.org/organisms/staphylococcus-aureus (accessed on 22 January 2022)). Moreover, PFGE was conducted by digesting genomic DNA using the *SmaI* enzyme (Takara Bio Inc., Shiga, Japan) according to a standard protocol of the Centers for Disease Control and Prevention (CDC, USA) [21], using a CHEF-MAPPER apparatus (Bio-Rad Laboratories, Hercules, CA), as described previously [22], and analyzed using the BioNumerics software (Applied Maths, Kortrijk, Belgium).

### 2.4. Statistical Analysis

The Statistical Package for the Social Sciences (SPSS) v.25 (IBM Corp., Armonk, NY, USA) was used for statistical analyses. Pearson’s chi-squared and Fisher’s exact test with Bonferroni correction were performed. Differences were considered significant at *p* < 0.05.

## 3. Results

### 3.1. Prevalence of S. aureus with Enterotoxingenes

**Figure 1:**
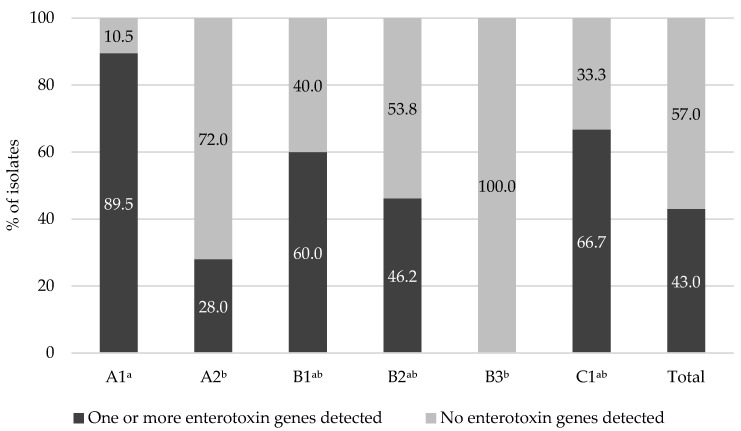
Prevalence of enterotoxin among 93 *Staphylococcus aureus* isolates from bulk tank milk in 6 dairy factories (A1 to C1). Values with different superscript letters represent significant differences by factories (*p* < 0.05).

Among the 93 *S. aureus* isolates, 40 (43.0%) carried at least 1 or more enterotoxin genes (Figure 1). However, the prevalence of enterotoxin genes was significantly high in *S. aureus* from factory A1 (89.5%), followed by factory C1 (66.7%), B1 (60.0%), and B2 (46.2%) (*p* < 0.05). Otherwise, the prevalence of enterotoxin genes in *S. aureus* from factory A2, owned by the same company as A1, was only 28.0%. Moreover, there was no *S. aureus*-carrying enterotoxin gene from factory B3, owned by the same company as B1 and B2.

### 3.2. Distribution of Virulence Genes

**Figure 2:**
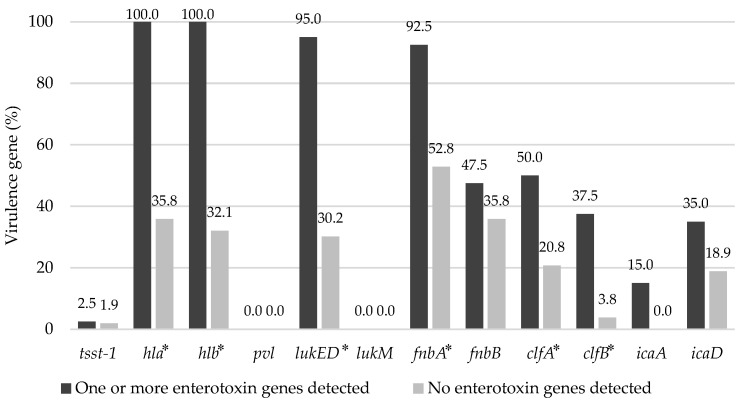
Distribution of virulence genes among 93 *Staphylococcus aureus* isolates from bulk tank milk in 6 dairy factories. * Asterisks indicate significant differences in the distribution of virulence genes depending on the presence of enterotoxin genes (*p* < 0.05).

The distribution of virulence genes in 93 *S. aureus* isolates is shown in Figure 2. The prevalence of virulence genes showed the difference depending on the presence of enterotoxin genes. In other words, *S. aureus*-carrying enterotoxin genes showed a higher prevalence in all virulence genes, except for *pvl* and *lukM*, which were not detected in any *S. aureus* isolates, than in the isolates without the enterotoxin genes. Among *S. aureus* isolates carrying the enterotoxin genes, *hla* (100.0%) and *hlb* (100.0%) were highly prevalent, followed by *lukED* (95.0%), *fnbA* (92.5%), *clfA* (50.0%), *fnbB* (47.5%), *clfB* (37.5%), *icaD* (35.0%), and *icaA* (15.0%). Moreover, the prevalence of six genes (*hla*, *hlb*, *lukED*, *fnbA*, *clfA,* and *clfB*) was significantly higher in *S. aureus*-carrying enterotoxin genes than in those without the enterotoxin genes (*p* < 0.05).

The distribution of virulence gene patterns in 40 *S. aureus* isolates carrying the enterotoxin genes is shown in Table 2. Although 19 virulence gene patterns showed no significant differences between the prevalence rate, *S. aureus* isolates carrying 8 virulence genes simultaneously were found in factory A1, which showed the highest prevalence of enterotoxin genes.

### 3.3. Genotypic Characteristics of S. aureus Carrying the Enterotoxin Genes

**Figure 3:**
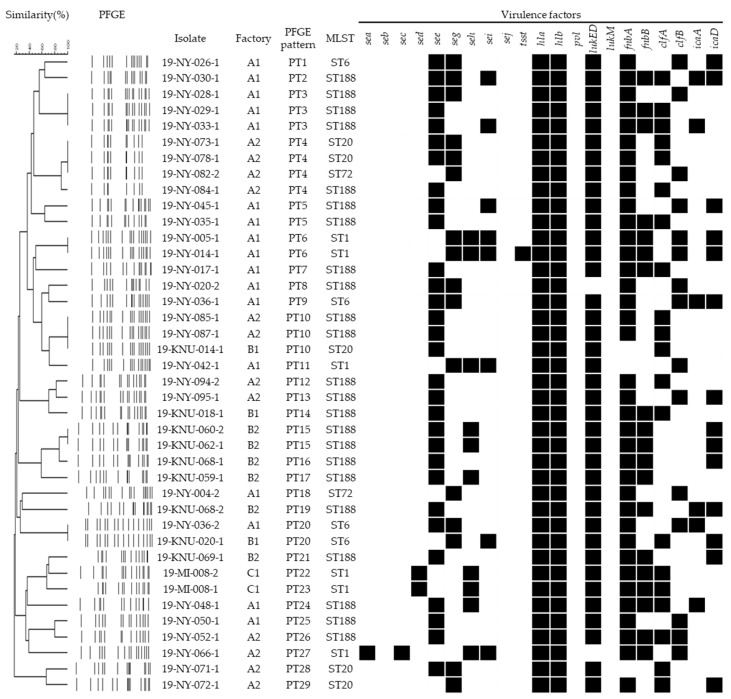
Phylogenetic dendrogram of PFGE patterns showing the relevance of the 40 enterotoxin-positive *Staphylococcus aureus* isolates from bulk tank milk in 6 dairy factories. *S. aureus* showing similarities of <85% in PFGE were considered to be unrelated.

The genetic relationship of 40 *S. aureus* isolates carrying the enterotoxin genes is shown in Figure 3. Although PFGE revealed 29 clusters showing 85% similarity and no correlation with virulence factors, 5 STs were associated with virulence genes. In particular, none of the isolates of ST1 and ST72 carried the *see* gene; however, all isolates of ST188 harbored the *see* gene. Additionally, all isolates of ST1 carried the *seh* gene, whereas isolates of ST6, ST20, and ST72 did not harbor the *seh* gene. One isolate with both *sea* and *sec* genes and two isolates with the *sed* gene were only revealed in ST1. Although the relationship between ST and virulence factors, except for the enterotoxin genes, was not characterized in this study, all STs harbored *hla* and *hlb* genes; however, ST72 did not harbor *fnbB*, *clfA*, *icaA,* and *icaD* genes.

## 4. Discussion

*S. aureus* produces many potential virulence factors to promote host tissue colonization, adhere to host cells, resist physical removal, invade host cells, and compete for iron and other nutrients [23]. In particular, plasmids and transposons typically contain antibiotic-resistance genes, whereas phage-related and pathogenicity islands contain most *S. aureus* toxins and other virulence determinants [24]. Moreover, most *S. aureus* toxins and virulence factors are encoded on *S. aureus* pathogenicity islands (SaPIs) [24], and the transfer of virulence genes via SaPIs may increase the risk of pathogenicity because they are transmitted not only to the same species but also to completely unrelated bacteria such as *L. monocytogenes.*

In Korea, five major dairy companies produce 84% of the total milk and dairy products (ATFIS, 2020) [25], and *S. aureus* isolated from six factories operated by three dairy companies was investigated in this study. Although 93 *S. aureus* isolates were from normal bulk tank milk, not from mastitis, 40 (43.0%) carried at least 1 or more enterotoxin genes. Moreover, the presence of enterotoxin genes was significantly different among the factories. Interestingly, even in the same company, the prevalence of enterotoxin genes showed a significant difference among the factories. Previous studies reported the presence of enterotoxins in 27.1–79.0% of *S. aureus* in milk and dairy products, and the frequency of enterotoxin genes varied by geographic region [13,16,26]. Furthermore, Schelin et al. (2011) [27] reported that enterotoxin production was influenced by environmental factors, such as temperature, pH, and moisture; therefore, management programs of dairy factories may affect the level of enterotoxins.

In the distribution of several virulence factors, which implicate the pathogenesis of *S. aureus*, all virulence genes, except *pvl* and *lukM* tested in this study, were shown to be present at a higher rate in the enterotoxin-producing isolates than in non-producing isolates. Primarily, *hla* and *hlb* genes allow for more persistence of pathogens in the mammary gland and cause chronic infection [6]. Therefore, these genes are the most prevalent in *S. aureus* from bovine mastitis milk [17,28,29,30]. Moreover, enterotoxin-producing *S. aureus* from normal bulk tank milk in this study is highly likely to cause chronic mastitis.

Leukocidin, including PVL, a type of cytotoxin, is an important factor contributing to increased virulence [31,32]. In this study, although none of the *S. aureus* isolates carried *pvl* and *lukM* genes, 95.0% of enterotoxin-producing isolates carried the *lukED* gene, and the prevalence was significantly higher than that in the non-producing isolates. Previous studies have reported that *lukED* was the most prevalent in *S. aureus* isolated from bovine mastitis milk in South Africa (100.0%), Finland (96.6%), Japan (96.0%), and the US (95.0%) [16,33,34,35]. The *lukED* has the ability to penetrate and kill cells, such as neutrophils that carry the bovine chemokine receptor, and is essential for the pathogenesis of mastitis [36]. Although *pvl* and *lukM*, which are associated with leukocyte destruction and necrosis and severity of mastitis, respectively, were not detected in any of the isolates, the high prevalence of *lukED* in enterotoxin-producing isolates could imply that there is a potential to induce mastitis.

Adhesion is essential to invade host cells and evade immune responses [37], and the biofilm-forming ability causes chronic or persistent infections [38]. *S. aureus* harbors various adhesion- and biofilm-related genes, such as *fnbA*, *fnbB*, *clfA*, *clfB*, *icaA*, and *icaD*, and the prevalence of these genes has been reported to vary according to geographic regions [18]. In this study, the prevalence of *fnbA* (92.5% vs. 52.8%), *clfA* (50.0% vs. 20.8%), and *clfB* (37.5% vs. 3.8%) genes between enterotoxin-producing and non-producing isolates was significantly different. Previous studies have also reported a high prevalence of *fnbA* in *S. aureus* isolated from mastitis [29,39].

Another superantigen virulence factor, *tsst-1*, hyperactivates the host immune response, resulting in toxic shock syndrome in humans, and can retain biological activity in milk after pasteurization [6]. Although each enterotoxin-producing and non-producing isolates carried *tsst-1* in this study, *S. aureus* carrying the *tsst-1* gene can lead to a public health concern.

In this study, five STs, ST1, ST6, ST20, ST72, and ST188, were revealed in enterotoxin-producing isolates. Song et al. (2015) [40] reported that ST1, ST6, and ST188 are frequently found to be associated with staphylococcal food poisoning in East Asia. Wang et al. (2018) [41] also reported that *S. aureus* ST188, a major lineage-causing infection in humans and livestock, possesses high nasal colonization and biofilm formation abilities in several host species. Mechesso et al. (2021) [42] have identified ST188 from bovine mastitis in Korea, but its prevalence was 23.3%. In this study, the prevalence of ST188 was higher than that of other STs; therefore, it seems to have a high potential to induce mastitis. Moreover, interestingly, all isolates in ST188 harbored the classical enterotoxin gene, *see*.

The *see* gene, which has the highest prevalence (75.0%) in enterotoxin-producing *S. aureus* in this study, has reported no or only low detection in milk [1,13,43]. Homsombat et al. (2021) reported that the growth of *see*-positive staphylococci in milk was significantly faster at a temperature of more than 8 °C. In this study, each bulk tank milk sample was collected from factories and sent to the laboratory under 4 °C. However, the bulk tank milk might not have been refrigerated to the correct temperature during transportation from the farms to the factories, or the temperature of whole milk might have risen during the milk in/out process.

Moreover, novel SEs, *seg*, *seh*, and *sei* genes were detected in 40.0%, 25.0%, and 20.0% of isolates, respectively, and have also been reported in food poisoning and bovine mastitis-related *S. aureus* worldwide [40,42,44,45]. Although the mechanism of novel enterotoxins in *S. aureus* is not clearly known, several molecular studies have suggested that they may play an important role in enhancing virulence because they are widely distributed in *S. aureus* [46].

## 5. Conclusions

These data provide that *S. aureus* isolated from normal bulk tank milk, not from mastitis, has a high prevalence of enterotoxins and that *S. aureus*, which produces enterotoxins, has various virulence factors simultaneously, posing a public health threat. Moreover, the high presence of enterotoxins in bulk tank milk usually may be due to a combination of poor hygiene, bad milking technique, refrigeration failure, and unsanitary milking equipment. Therefore, developing a strong monitoring and sanitation program for dairy factories is important for hygienic milk production.

## Figures and Tables

**Table 1:** Primers used in this study.

Target	Sequence (5′ → 3′)	Size (bp)	References
*nuc*	F: GCGATTGATGGTGATACGGTT	279	[12]
	R: AGCCAAGCCTTGACGAACTAAAGC		
*sea*	F: GAAAAAAGTCTGAATTGCAGGGAACA	560	[13]
	R: CAAATAAATCGTAATTAACCGAAGGTTC		
*seb*	F:ATTCTATTAAGGACACTAAGTTAGGGA	404	[13]
	R: ATCCCGTTTCATAAGGCGAGT		
*sec*	F:CTTGTATGTATGGAGGAATAACAAAACATG	275	[13]
	R: CATATCATACCAAAAAGTATTGCCGT		
*sed*	F:GAATTAAGTAGTACCGCGCTAAATAATATG	492	[13]
	R: GCTGTATTTTTCCTCCGAGAGT		
*see*	F: CAAAGAAATGCTTTAAGCAATCTTAGGC	482	[13]
	R: CACCTTACCGCCAAAGCTG		
*seg*	F:TCTCCACCTGTTGAAGG	323	[13]
	R: AAGTGATTGTCTATTGTCG		
*seh*	F: CAATCACATCATATGCGAAAGCAG	376	[13]
	R: CATCTACCCAAACATTAGCACC		
*sei*	F: GTACCGTTGAAAATTCAG	461	[13]
	R: AGGCAGTCCATCTCCTG		
*sej*	F: TCAGAACTGTTGTTCCGCTAG	138	[13]
	R: GAATTTTACCAYCAAAGGTAC		
*tst*	F: CTGGTATAGTAGTGGGTCTG	271	[14]
	R: AGGTAGTTCTATTGGAGTAGG		
*hla*	F: CTGATTACTATCCAAGAAATTCGATTG	209	[15]
	R: CTTTCCAGCCTACTTTTTTATCAGT		
*hlb*	F: GTGCACTTACTGACAATAGTGC	309	[15]
	R: GTTGATGAGTAGCTACCTTCAGT		
*lukS*/*F*-PV	F: ATCATTAGGTAAAATGTCTGGACATGATCCA	433	[15]
	R: GCATCAASTGTATTGGATAGCAAAAGC		
*lukED*	F: TGAAAAAGGTTCAAAGTTGATACGAG	269	[16]
	R: TGTATTCGATAGCAAAAGCAGTGCA		
*lukM*	F: TGGATGTTACCTATGCAACCTAC	780	[15]
	R: GTTCGTTTCCATATAATGAATCACTAC		
*fnbA*	F: GTGAAGTTTTAGAAGGTGGAAAGATTAG	643	[17]
	R: GCTCTTGTAAGACCATTTTTCTTCAC		
*fnbB*	F: GTAACAGCTAATGGTCGAATTGATACT	524	[17]
	R: CAAGTTCGATAGGAGTACTATGTTC		
*clfA*	F: ATTGGCGTGGCTTCAGTGCT	292	[18]
	R: CGTTTCTTCCGTAGTTGCATTTG		
*clfB*	F: ACATCAGTAATAGTAGGGGGCAAC	205	[18]
	R: TTCGCACTGTTTGTGTTTGCAC		
*icaA*	F: CCTAACTAACGAAAGGTAG	1315	[19]
	R: AAGATATAGCGATAAGTGC		
*icaD*	F: AAACGTAAGAGAGGTGG	381	[19]
	R: GGCAATATGATCAAGATAC		

**Table 2:** The virulence gene patterns of 40 enterotoxin-positive *Staphylococcus aureus* isolates from bulk tank milk in 6 dairy factories.

Virulence Gene Patterns	No. (%) of Isolates ^a^	Factory (No. of Isolates)
*hla*, *hlb*, *clfA*, *lukED*	2 (5.0)	A2 (1), B1 (1)
*hla*, *hlb*, *clfB*, *lukED*	1 (2.5)	A1 (1)
*hla*, *hlb*, *fnbA*, *clfB*	1 (2.5)	A1 (1)
*hla*, *hlb*, *fnbA*, *fnbB*, *clfB*	1 (2.5)	A2 (1)
*hla*, *hlb*, *fnbA*, *clfA*, *lukED*	6 (15.0)	A2 (6)
*hla*, *hlb*, *fnbA*, *clfB*, *lukED*	4 (10.0)	A1 (3), A2 (1)
*hla*, *hlb*, *fnbA*, *fnbB*, *lukED*	1 (2.5)	B2 (1)
*hla*, *hlb*, *fnbA*, *clfA*, *icaD*, *lukED*	2 (5.0)	A2 (1), B1 (1)
*hla*, *hlb*, *fnbA*, *clfB*, *icaA*, *lukED*	1 (2.5)	A1 (1)
*hla*, *hlb*, *fnbA*, *clfB*, *icaD*, *lukED*	3 (7.5)	A1 (2), A2 (1)
*hla*, *hlb*, *fnbA*, *fnbB*, *clfA*, *lukED*	6 (15.0)	A1 (3), B1 (1), C1 (2)
*hla*, *hlb*, *fnbA*, *fnbB*, *icaD*, *lukED*	4 (10.0)	B2 (4)
*hla*, *hlb*, *fnbA*, *clfB*, *icaA*, *icaD*, *lukED*	1 (2.5)	A1 (1)
*hla*, *hlb*, *fnbA*, *fnbB*, *clfA*, *icaA*, *lukED*	2 (5.0)	A1 (2)
*hla*, *hlb*, *fnbA*, *fnbB*, *icaA*, *icaD*, *lukED*	1 (2.5)	B2 (1)
*hla*, *hlb*, *fnbA*, *fnbB*, *clfA*, *clfB*, *lukED*	1 (2.5)	A2 (1)
*hla*, *hlb*, *fnbA*, *fnbB*, *clfB*, *icaD*, *lukED*	1 (2.5)	A1 (1)
*hla*, *hlb*, *fnbA*, *fnbB*, *clfA*, *icaA*, *icaD*, *lukED*	1 (2.5)	A1 (1)
*hla*, *hlb*, *fnbA*, *fnbB*, *clfB*, *icaD*, *lukED*, *tsst-1*	1 (2.5)	A1 (1)

^a^ No significant differences (*p* < 0.05).

## Data Availability

Not applicable.
